# Integrative machine learning reveals hidden and emerging co-regulatory gene networks for multi-phase glioblastoma outcome prediction

**DOI:** 10.1016/j.ejca.2025.116197

**Published:** 2025-12-24

**Authors:** Md Tamzid Islam, Fengwei Yang, Stephan Komladzei, Murshalina Akhter, Mihaela E. Sardiu, Yanming Li

**Affiliations:** a Department of Biostatistics & Data Science, University of Kansas Medical Center, 3901 Rainbow Boulevard, Kansas City, KS 66160, United States; b University of Kansas Cancer Center, 3901 Rainbow Boulevard, Kansas City, KS 66160, United States; c Kansas Institute for Precision Medicine, University of Kansas Medical Center, 3901 Rainbow Boulevard, Kansas City, KS 66160, United States

**Keywords:** Differential gene expression analysis, Glioblastoma, Network linear discriminant analysis, Multiple traits, Pathway enrichment, Protein-protein interaction, Kaplan-Meir survival comparison

## Abstract

**Background:**

Glioblastoma (GBM) is a highly prevalent and aggressive type of brain tumor characterized by profound molecular complexity and poor prognosis. While conventional biomarker studies focus on highly significant genes or proteins associated with cancer outcomes, the contribution of gene–gene co-regulation to GBM progression remains unclear.

**Methods:**

This study employs an application of integrative machine learning approach, utilizing a high-dimensional transcriptomic profile that considers gene-gene co-regulations to identify predictive gene networks involved in GBM occurrence and 1-year survival prediction. We further integrate these network models with both empirical protein-protein interaction (PPI) data and random walk-based information flow analysis across the PPI landscape.

**Results:**

This dual-layered approach uncovers gene modules that bridge transcriptional co-regulation with functional connectivity at the protein level. This integration highlighted several hub genes, including both strong and weak (e.g. BSN, RHOC, ANXA1, CSF1R, and ITGAM), that emerged as key molecular connectors involved in critical GBM processes such as immune response and neuronal signaling. Notably, these hub genes also exhibited cross-disease associations with traits including gut microbiome composition, type 2 diabetes, coronary artery disease, and other cancers, underscoring their systemic biological relevance.

**Conclusion:**

Overall, our findings through the computational approach underscore the significance of co-regulatory gene networks in GBM biology. It also demonstrates how integrating transcriptomic and protein-level interactions can refine prognostic modeling, advance biomarker discovery, and inform future therapeutic development.

## 1. Introduction

Glioblastoma multiforme (GBM) is one of the most common and aggressive brain tumors affecting the glial cells of the central nervous system, classified by WHO as a grade IV glioma due to its rapid growth and malignant nature [1]. It invades adjacent areas of the brain but rarely spreads outside the brain, which complicates early diagnosis and overall health outcomes, including life expectancy [2, 3]. Without prompt diagnosis and intervention, the prognosis is particularly poor, with survival averaging around 5 months [4]. Despite general treatment intervention median survival rate is approximately 12–15 months [5].

Researchers are continually exploring areas of advanced imaging techniques, stem cell research etc., to gain deeper insights into disease progression and develop improved diagnostic and therapeutic strategies [6, 7]. Recent studies have emphasized the importance of bioinformatics-driven workflow frameworks for extracting clinically meaningful insights from complex datasets, reinforcing the value of integrative computational approaches in biomarker discovery [8]. In this context, gene expression (GE) analysis has become fundamental to molecular biology, significantly advancing our understanding of complex biological systems. This is especially true in cancer research, including studies of glioblastoma (GBM), where GE analysis is used to identify potential biomarkers [9]. The advent of RNA sequencing (RNA-Seq) has revolutionized the field, enabling precise quantification of gene expression changes under various biological conditions. To better address the complexity and high dimensionality of RNA-Seq data, researchers are increasingly integrating machine learning (ML) alongside traditional analytical methods. This integration helps uncover hidden patterns and relationships to identify potential biomarkers and hub genes associated with cancer development and progression [10]. While applying ML methods, researchers often use the top differentially expressed (DE) genes (marginally more informative or strong) as potential features that lead to the identification of hub genes only from the top DE genes [11]. The contribution of genes that provide slightly less useful (weaker) information is typically overlooked. However, recent studies suggest that cancer onset and progression are driven not only by changes in individual gene expression but also by alterations in gene interactions [12]. Specifically in the context of glioblastoma, gene co-expression networks highlight how gene–gene interactions contribute to tumor heterogeneity and progression [13]. These network-level connections can often be validated through their corresponding protein–protein interactions, revealing how transcriptional co-regulation translates into molecular interactions at the protein level [14]. Therefore, previously overlooked weak genes for ML models may play a crucial role by co-regulating with stronger genes. Recent advances in genomic, transcriptomic, and multi-omics integration approaches have demonstrated the value of network-based analyses in revealing regulatory and disease-relevant molecular interactions [15]. These studies highlight how integrative frameworks can capture complex biological processes that are often missed in single-layer or single-gene analyses. In 2019, Li et.al. proposed a network-based machine learning model that can consider both marginally informative (strong) and less informative (weak) differentially expressed genes in outcome prediction. This model leverages inter-feature correlations for variable screening of ultrahigh-dimensional features. It was also assessed as well-performing when compared to other competing models for classifying the outcome. Therefore, the model can identify several gene networks, aiding in the study of not only a single hub gene but also other associated genes, thus contributing to better prediction of cancer outcomes [16].

In this study, we introduce an integrative framework combining netLDA-based gene network discovery with protein–protein interaction (PPI) validation to identify biologically meaningful hub genes in glioblastoma outcome prediction. The network-based machine learning model (netLDA) utilizes both strong and weak genes, leveraging their gene coregulations, to identify potential gene networks in predicting GBM disease occurrence and 1-year survival, which are key outcomes of clinical relevance [17, 18]. Building on the original netLDA approach, we enhance biological interpretability by integrating PPI network analysis to validate and refine these machine learning–derived gene networks. Notably, our framework also reveals the relationship between predicted gene networks that appear unconnected in conventional PPI databases but show functional associations through random walk–based information flow analysis. These allow us to identify hub genes, key molecular connectors that bridge transcriptional co-regulation with protein-level interaction networks. Finally, we investigate the biological pathways and disease traits associated with the identified gene networks, along with a comparison of survival curves, providing a comprehensive view of the underlying biological mechanisms.

## 2. Materials and methods

### 2.1. Experimental data

We employed two databases, the Cancer Genome Atlas (TCGA), which consists of GBM patient datasets, and the Genotype-Tissue Expression (GTEX), which consists of Non-disease datasets, using the University of California Santa Cruz (UCSC) Xena (Supplementary Figure 1) [19]. RNA-Seq data with RSEM expected gene counts and patient survival data are obtained from the TCGA database. The data were harmonized for TCGA-GTEx samples, including batch-effect correction. The dataset comprised 1351 samples, including 153 GBM patients (Primary Tumor) and 1148 Non-disease samples used in the disease/Non-disease model for GBM prediction. To perform a 1-year survival prediction model, the gene data of the 153 GBM patients were used. Out of the total patients, 71 survived for at least one year, and 60 patients died before completing one year. The remaining patients were censored and not included in the analysis. Both models used 18328 genes mapped using the “annotationDBI” R package.

### 2.2. Statistical methods

All analyses were performed in R version 4.4.1. Differential expression analysis was conducted using edgeR, where lowly expressed genes were removed with “filterByExpr”, library size differences were normalized using TMM “calcNormFactors”, and a quasi-likelihood negative binomial model “estimateDisp”, “glmQLFit” was used for inference. Strong and weak genes were then defined based on ranked differential expression metrics. netLDA was applied to predict GBM occurrence and one-year survival using a correlation threshold of 0.9, maximum network component size of 3, and 5-fold cross-validation for optimization (Supplementary Table 1). Kaplan–Meier survival curves and log-rank tests were generated using the survival and “ggsurvplot” packages. Venn diagrams were created using “ggVennDiagram”, and ROC curves were produced using “ROSE”. Protein–protein interactions for the identified networks were evaluated from STRING using the “STRINGdb” package.

### 2.3. Gene enrichment pathway, network analysis, explanation of genes, and multiple trait analysis

The gene enrichment analysis for pathway observation was conducted using Consensus Path DB (http://cpdb.molgen.mpg.de). The interaction network for nonconnected networks from STRING output was observed using ITM Probe, where we used the random walk method with information flow in protein-protein interaction and selected the channel model to find the most likely paths between the given sources (i.e., strong genes) and sinks (i.e., weak genes). Homo sapiens BioGRID was selected as interaction graph with default termination probability 0.15 and maximum shown network 40 (https://www.ncbi.nlm.nih.gov/CBBresearch/Yu/mn/itm_probe/). The biological significance of the genes in our detected networks is found in GeneCards (https://www.genecards.org/). Multiple traits are obtained from genome-wide association studies (https://www.ebi.ac.uk/gwas/).

**Fig. 1.**
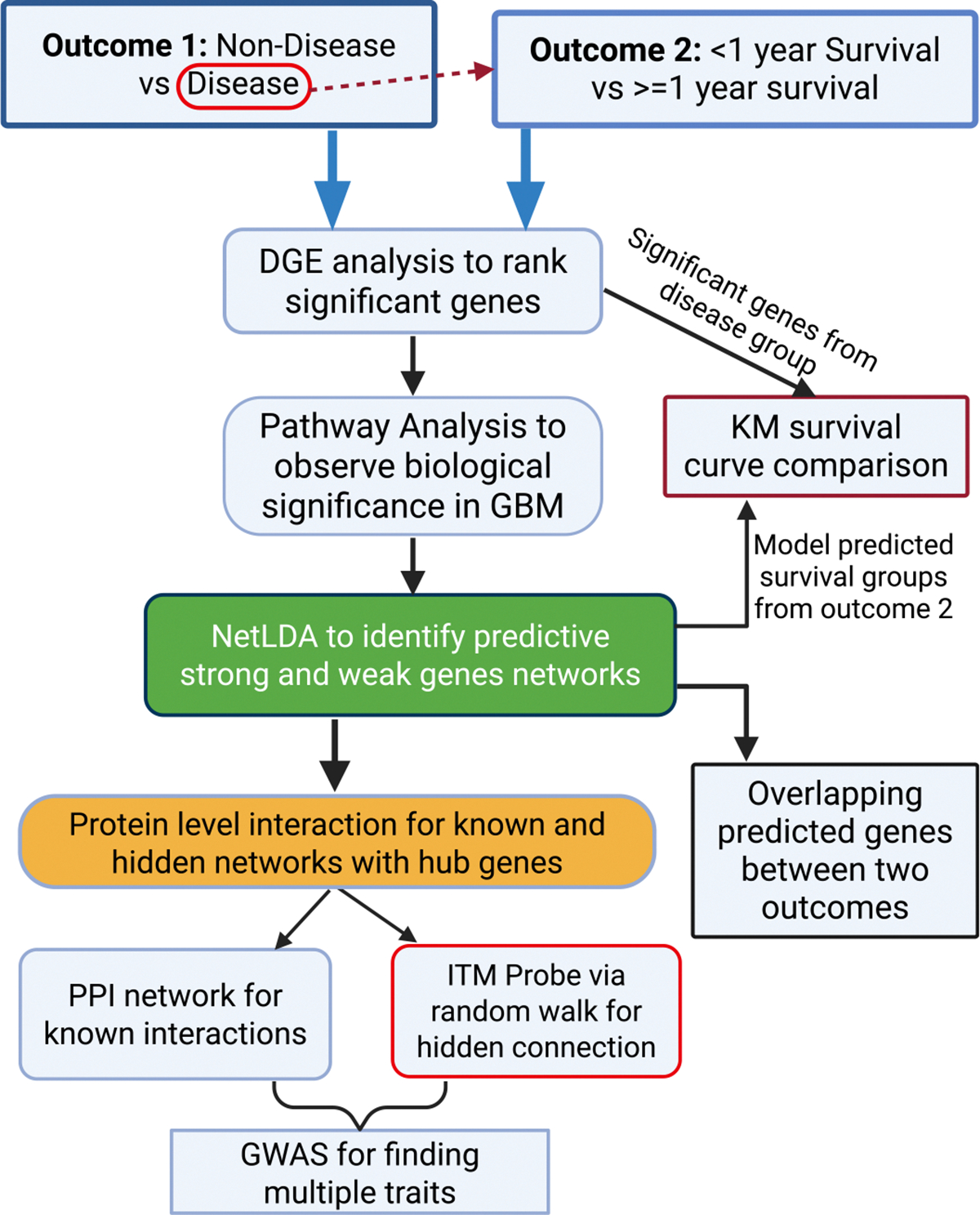
Workflow for identifying hidden and emerging co-regulatory gene networks for multi-phase glioblastoma outcome prediction.

The above discussed methodology is demonstrated in Fig. 1 through a workflow

## 3. Result and analysis

### 3.1. Identifying and ranking DEGS

**Fig. 2.**
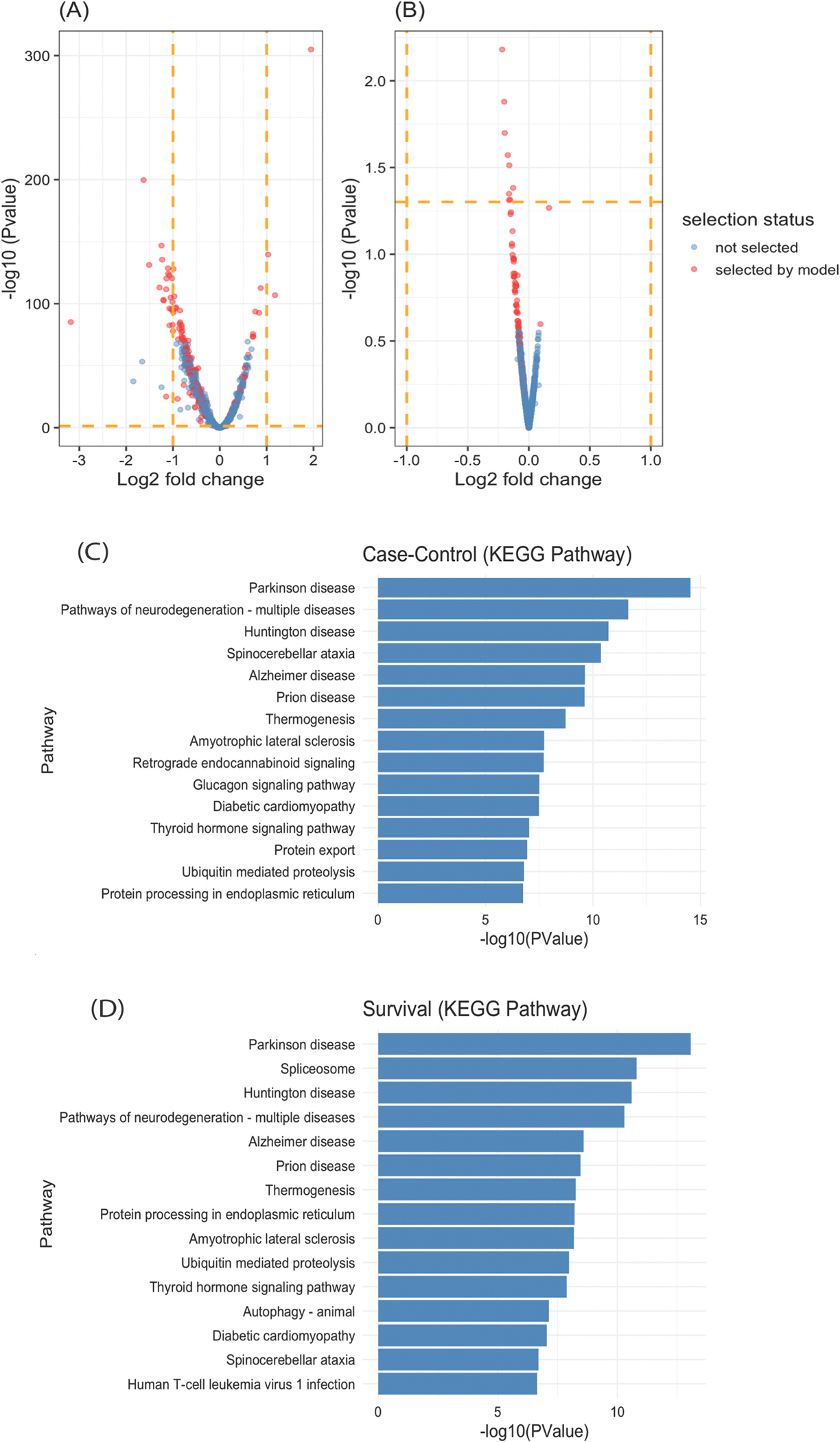
Overall gene summary from differentially expressed genes analysis using edgeR along with enriched pathways. (A) Comparative Analysis of Disease and Non-Disease States (B) Analysis of One-Year Survival Outcomes. In this visualization, dots positioned above the horizontal yellow line signify genes with a p-value < 0.05, highlighting those that are significantly differentially expressed. The vertical yellow lines represent critical log_2_ fold change thresholds of + 1 and −1, serving as key markers for gene expression variation. Red dots denote genes that have been identified and selected by our analytical model, whereas blue dots represent genes that were not selected. (C-D) An overview of the top 15 KEGG pathways associated with the Disease vs. Non-Disease analysis, as well as those linked to the One-Year Survival analysis, underscores the biological processes and pathways that are most significantly impacted in our study.

Our study focuses on two main outcomes: predicting cancer and predicting one-year survival (Supplementary Tables 2 and 3). We began by analyzing differentially expressed genes (DEGs) associated with both outcomes. To achieve this, we filtered out genes with low expression levels, specifically those with counts below 10 in more than 70 % of the samples, using the edgeR software. This process resulted in 4662 genes for the disease prediction model and 4605 for the survival prediction model. Next, we utilized the log2 fold change and p-values of these differentially expressed genes to identify sets of upregulated and downregulated genes. After further refining our selection based on smaller p-values, we chose the top differentially expressed genes for the netLDA model. This model classified genes into strong and weak groups. In Fig. 2A and 2B, we visualized the distribution of significant upregulated (>1 vertical dashed line) and downregulated (<1 vertical dashed line) genes, with significance indicated above the horizontal dashed line, for both models. Our model highlighted the red genes, while the blue genes were not selected.

### 3.2. Gene enrichment and pathway analysis

We then performed pathway analysis to understand the biological significance of the genes in our study, both in disease vs non-disease and survival model. Fig. 2C illustrates the top 15 significantly enriched KEGG pathways for disease and non-disease conditions. This analysis demonstrated that Parkinson’s disease is the most significantly enriched KEGG pathway, followed by Pathways of neurodegeneration-multiple disease and Huntington’s disease. These conditions are all associated with neurodegeneration and lead to dysfunction in nerve cells. The survival prediction analysis highlights the significant enrichment of the top 15 KEGG pathways, as illustrated in Fig. 2D. Within these pathways, Parkinson’s disease emerges as the most enriched, a result consistent with the disease and non-disease conditions. Furthermore, Huntington’s disease ranks among the top three enriched KEGG pathways. These diseases are related to GBM through neurodegenerative processes reflecting the biological importance of our selected genes.

### 3.3. Model implementation and performance evaluation

To evaluate the overall performance, we utilized the differentially expressed genes identified by edgeR in conjunction with the netLDA model for predictive analysis. This analysis aimed to predict binary outcomes, specifically distinguishing between disease and non-disease states, as well as survival outcomes of less than one year compared to one year or longer. We began by selecting a group of 50 genes that were highly differentially expressed, which we referred to as “strong genes.” For each of these strong genes, we treated it as an anchor gene and identified highly correlated genes (with a Pearson correlation greater than 0.9) associated with it. To ensure manageability, we limited the maximum size of each connected component to 3 genes. We kept these parameters consistent while conducting a 5-fold cross-validation of the netLDA model to identify the optimal model parameters. We specifically fine-tuned the number of marginally informative genes (the set of strong genes) and marginally uninformative but jointly informative genes (the set of weak genes) to optimize our model. We then applied the netLDA model to both outcomes using the selected sets of strong and weak genes. To evaluate the model’s performance, we used the Receiver Operating Characteristic (ROC) curve and found that it exhibited high performance, achieving an Area Under the Curve (AUC) of 0.999 for Disease/Non-Disease prediction (Fig. 4A) and 0.7985 for 1-year survival prediction (Supplementary Figure 2). In addition to the 50 selected strong genes for both outcomes, our model identified 147 weak genes for the Disease/Non-Disease model and 30 weak genes for the survival prediction model. The network-adjusted difference estimate (DE), along with their log fold changes and marginal p-values, is presented in Supplementary Tables 4 and 5.

### 3.4. Detection of top-selected genes and predictive gene networks

#### 3.4.1. Top genes based on network adjusted difference estimate (DE)

**Fig. 3.**
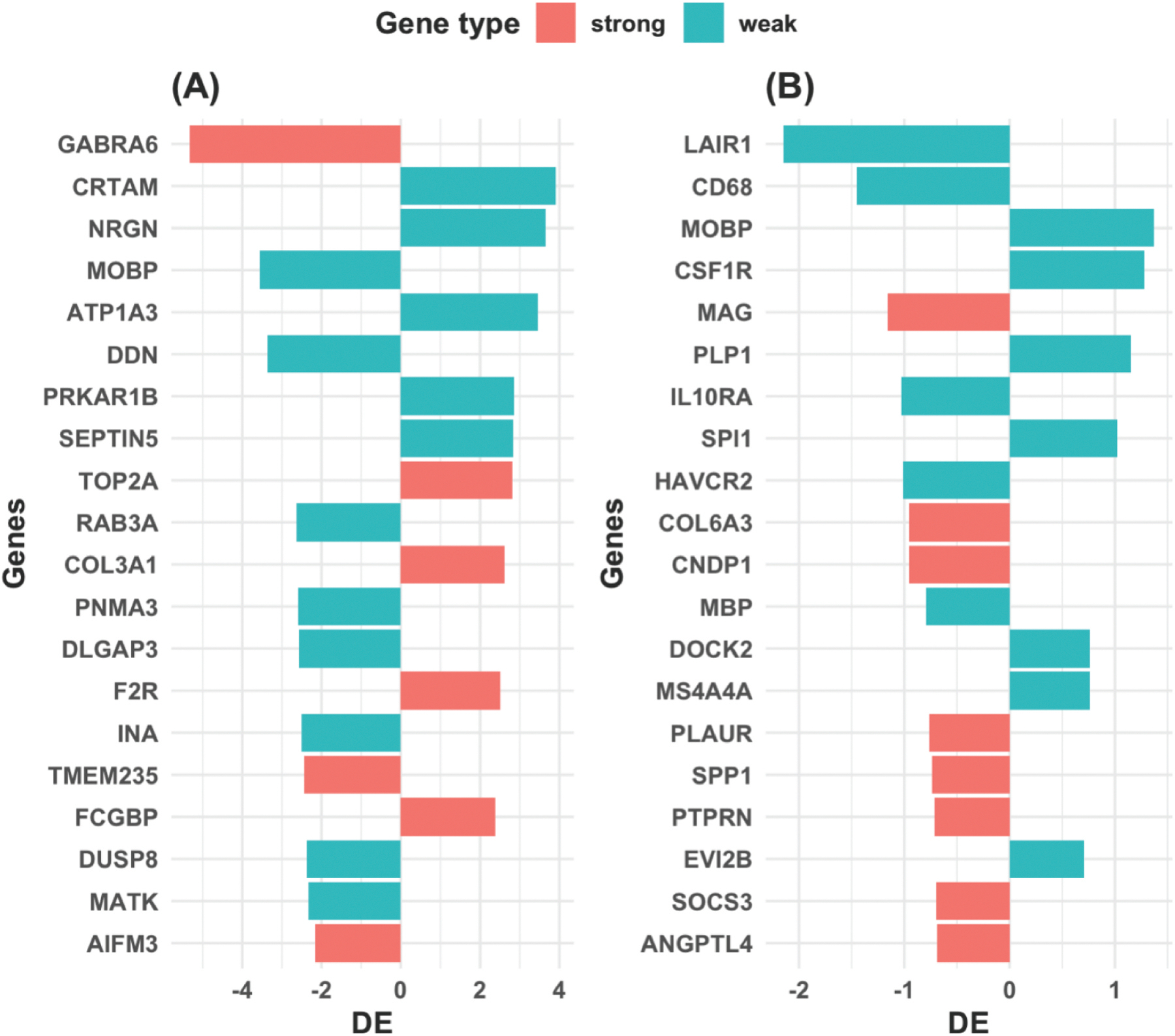
The top 20 selected genes were identified based on their Difference Estimate (DE) values, which highlight distinctions between strong and weak gene expressions. This analysis includes two key components: (A) a detailed examination of gene expressions in relation to disease versus non-disease states, and (B) an assessment of these genes in the context of 1-year survival outcomes in affected individuals.

The netLDA model has identified several gene networks that can predict glioblastoma (GBM) disease and 1-year survival status. From these gene networks, we can identify the top genes for prediction based on the absolute values of the network-adjusted difference estimates (DE). Fig. 3A illustrates the top 20 genes for each model, along with their classification as strong or weak predictors. In the disease prediction model, the top five strong and weak genes, along with their respective DE values, are as follows: GABRA6 (strong, DE = −5.33), CRTAM (weak, DE = 3.91), NRGN (weak, DE = 3.65), MOBP (weak, DE = −3.65), ATP1A3 (weak, DE = 3.46) (Supplementary Table 4). For the 1-year survival prediction model (shown in Fig. 3B), the top five strong and weak genes are: LAIR1 (weak, DE = −2.14), CD68 (weak, DE = −1.45), MOBP (weak, DE = 1.37), CSF1R (weak, DE = 1.28) and MAG (strong, DE = −1.16) (Supplementary Table 5). According to Table 1, we can conclude that all the genes in the disease prediction model are marginally significant at the 5 % level. In contrast, in the 1-year survival prediction model, most of the genes are found to be marginally insignificant. This highlights the different contributions of weak genes compared to strong genes in predicting survival outcomes.

#### 3.4.2. Functional and protein interaction insights of the gene networks

**Fig. 4.**
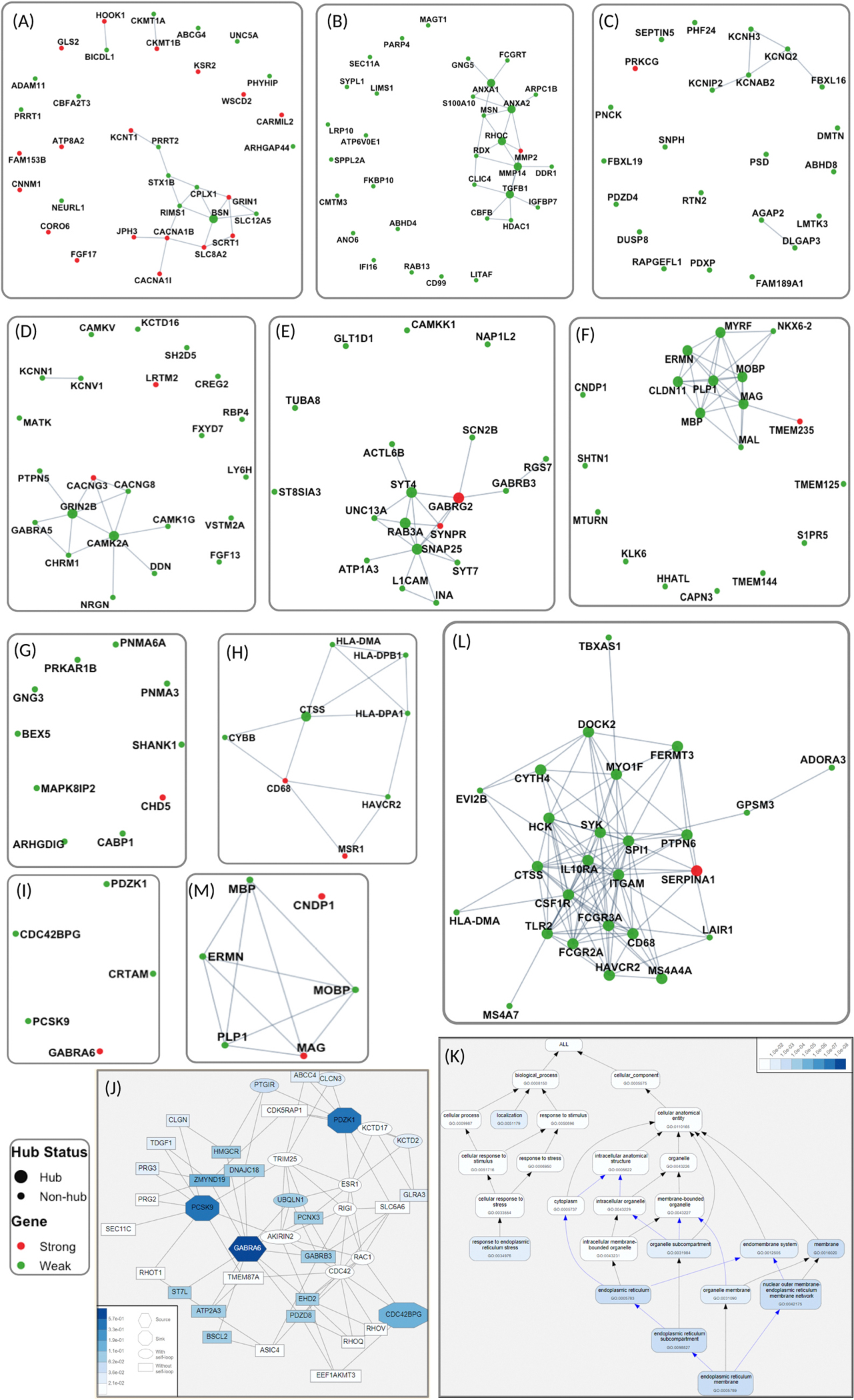
Protein-Protein interaction for the predictive gene networks and hidden information from the genes not connected at the protein level. (A-I) Illustrates interactions for the disease/non-disease model. (J) Shows the interaction of 18 genes from our network with other genes (not included in our model) using the ITM probe. (K) Provides biological enrichment analysis for the genes from network 18, as well as those connected to it. (L-M) Focuses on interactions within the 1-year survival prediction model. The lines connecting the circles represent their interactions based on various experimental studies. Red circles denote strong genes, while green circles indicate weak genes.

The gene networks predicted by our models are presented in Table 1, accompanied by Supplementary Figures 3(A-B) and 4, which are presented in the form of heatmaps. These heatmaps illustrate similar expression patterns across each network. Our disease prediction model identified nine predictive gene networks, each containing at least five genes (see Table 1). The largest network comprises 36 genes, which include 19 strong genes and 17 weak genes. The other networks primarily consist of weak genes, with only one or two strong genes in each. Upon reviewing the protein-protein interaction plots in Fig. 4 for each network, we identified the connections among the genes at the protein level. A gene is categorized as a hub gene if it interacts with five or more other genes. In network 2 (Fig. 4A), BSN emerges as a central hub tied to presynaptic cytoskeletal architecture in neurons, reflecting a core organizational axis of synaptic connectivity. The composition of network 26 (Fig. 4B) reveals a constellation of functionally intertwined genes—RHOC, TGFB1, MMP14, ANXA2, and ANXA1—collectively mapped to central neuronal processes and brain activity. Extending this mechanistic landscape, network 21 (Fig. 4D) highlights CAMK2A as a pivotal player in synaptic plasticity, belonging to the Ca(2+)/calmodulin-dependent kinase family and orchestrating dynamic glutamatergic signaling.

Network 12 (Fig. 4E) is characterized by pronounced modularity, with a strong hub surrounded by three supporting nodes—GABRG2, RAB3A, SYT4, and SNAP25—each underpinning essential stages of synaptic vesicle docking, membrane fusion, and neurotransmitter exocytosis. Despite its small size, network 28 (Fig. 4F) is saturated with myelin-related hub genes (MAG, ERMN, MBP, PLP1, CLDN11, MOBP, and MYRF), offering a structurally cohesive view of myelination and axonal insulation within neural tissue. Network 17 (Fig. 4H) shows CTSS as a solitary hub, with the broader module converging on two robustly interconnected immune regulators, MSR1 and CD68.

Conversely, network 15 (Fig. 4C) is defined by the absence of a dominant hub, illustrating distributed connectivity patterns that may support more diffuse regulatory roles. Notably, several networks including 18 (Fig. 4I) appear devoid of overt protein-level connections via STRING, presenting as sets of seemingly isolated proteins. However, leveraging random walks in protein-protein interaction space using ITM Probe analysis uncovers hidden information flow and indirect regulatory bridges. These pathways converge on molecular signatures of endoplasmic reticulum (ER) stress, as revealed through biological enrichment (Fig. 4J and 4K), highlighting the power of advanced network analytics to expose hidden functional associations beyond direct physical interactions.

In our 1-year survival prediction model, we identified two significant predictive gene networks, each consisting of more than five genes. In network 41, there are a total of 26 genes: one strong gene and 25 weak genes. Although individually the weak genes may not provide much information, together they contribute significantly to survival prediction. The protein level structures illustrated in Fig. 4L are highly interconnected, indicating strong relationships among the genes at the protein level. The strong gene identified is SERPINA1, a serine protease inhibitor linked to chronic obstructive pulmonary disease, emphysema, and chronic liver disease. Among the weak genes, several are co-regulated, including MS4A4A, CD68, HAVCR2, PTPN6, SYK, ITGAM, CSF1R, FCGR3A, TLR2, FCGR2A, CTSS, SPI1, IL10RA, HCK, FERMT3, MYO1D, DOCK2, CYTH4, and PTPN6. In network 3, although none of the genes are connected to five others, there are strong connections among most of them. Specifically, ERMN, MAG, MOBP, PLP1, and MBP are interconnected, except CNDP1, as shown in Fig. 4M.

### 3.5. Survival curves comparison of top DE genes

**Fig. 5.**
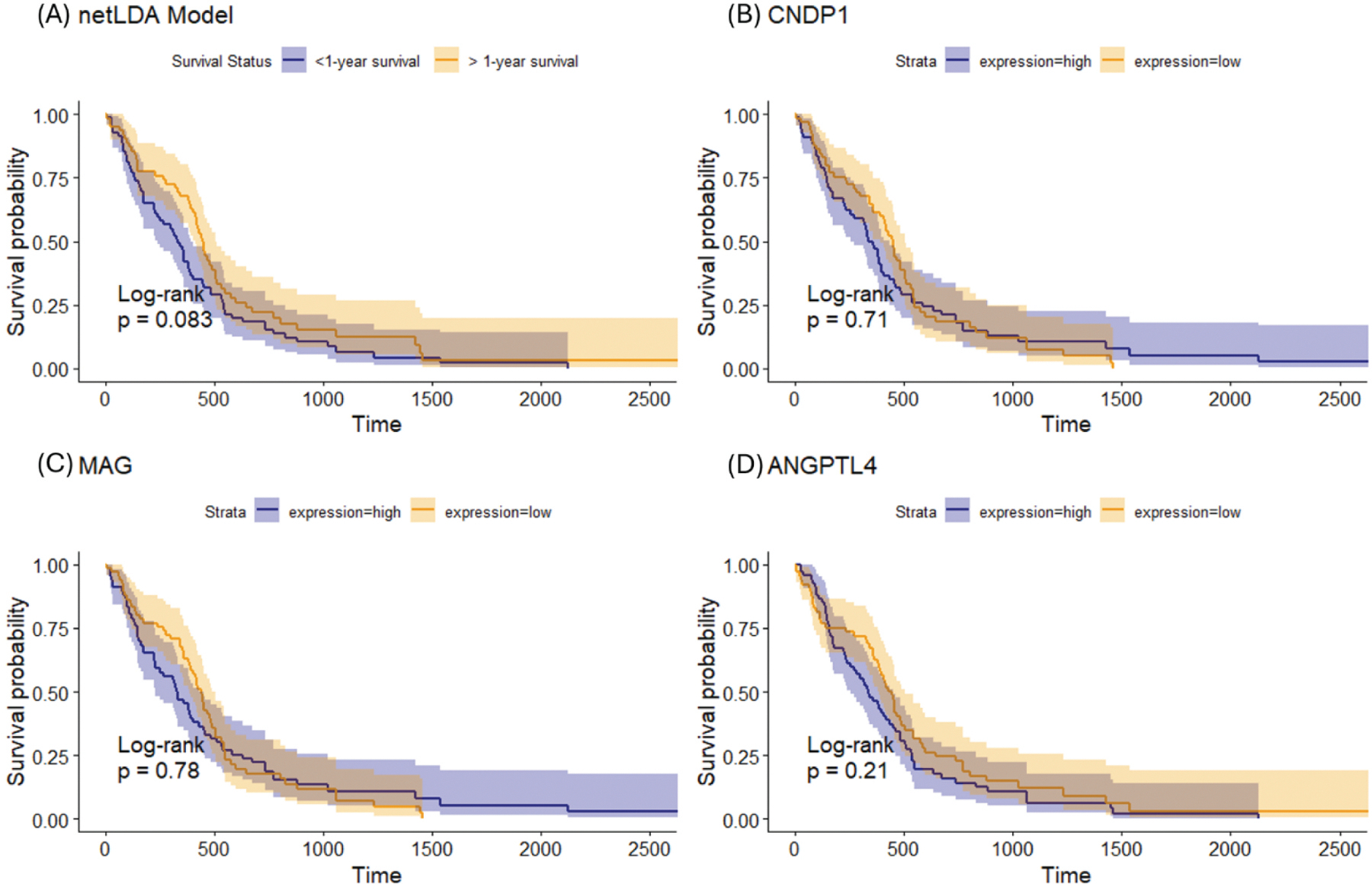
Kaplan-Meier curves and log-rank test results. (A) The KM curves and log-rank test compare two groups predicted by netLDA: long survival (≥ 1 year) and short survival (< 1 year). (B-D) The curves and log-rank tests assess high- and low-expression levels of the top selected strong genes, based on median expression values.

After exploring the biological relevance of the identified networks through protein-level interactions, we further evaluated the model’s ability to capture prognostic information by comparing survival outcomes. We fitted Kaplan-Meier (K-M) survival curves between the two predicted patient groups (<1 year and >= 1 year). For additional benchmarking, we generated K-M curves for patients stratified by high versus low expression of select top network-adjusted differentially expressed (DE) genes that demonstrated marginal significance (Fig. 5). We selected three prominent genes—MAG, CNDP1, and ANGPTL4 that had marginal p-values significant at the 5 % significance level. After fitting the survival curves, we found that our model was able to separate the survival groups more effectively (p-value = 0.083) than any individual strong gene, such as CNDP1 (p-value = 0.71), MAG (p-value = 0.78), or ANGPTL4 (p-value = 0.21), as determined by the log-rank test. The survival curves of these three genes did not separate well compared to our model. This indicates that our model can integrate information from both strong and weak genes to more effectively differentiate between patients’ survival groups than the separation based solely on high and low expression of a single gene.

### 3.6. Overlapping genes in both models

We next examined the common genes present in both outcome models (Supplementary Figure 5). In this analysis, only two genes are shared between the strong gene sets of the disease/non-disease model and the survival model: CHI3L2 and CD163. In contrast, there are seven overlapping genes between the weak gene sets of both models. These genes are MOBP, PLP1, MBP, CTSS, HAVCR2, ERMN, and HLA-DMA. Notably, CHI3L2 and CD163 are not connected to other genes, as seen in the top predictive networks. However, MBP, MOBP, ERMN, and PLP1 are part of the same network in both models. Additionally, in the survival model, CTSS, HAVCR2, and HLA-DMA form another network, while CTSS and HAVCR2 are connected within the same network in the disease prediction model. Therefore, the identification of these overlapping genes between the two models emphasizes their potential as robust biomarkers that link disease risk with patient prognosis.

### 3.7. Gene analysis related to multiple traits using genome-wide association studies (GWAS)

**Fig. 6.**
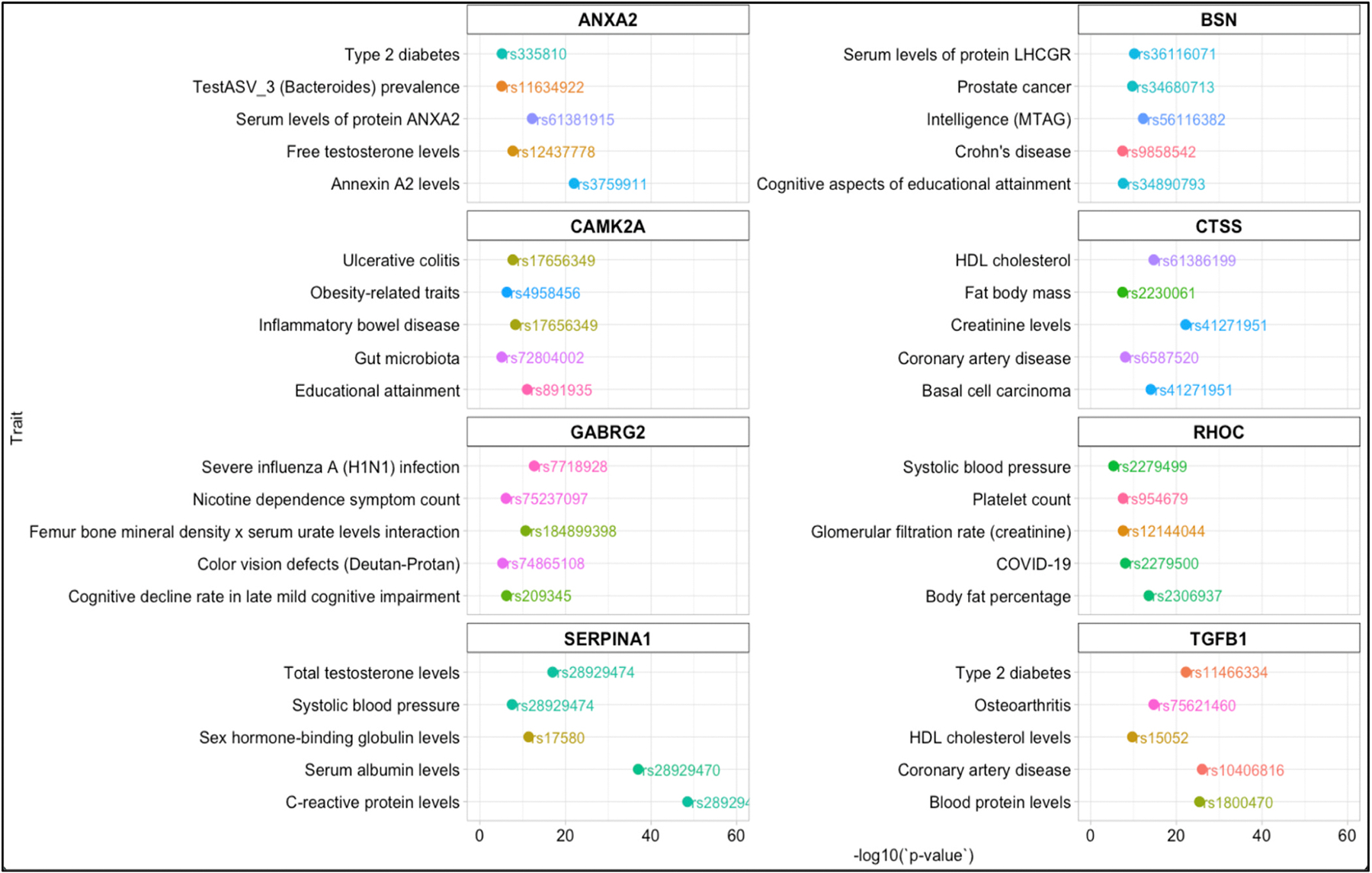
Traits and their variants for selected hub genes are reported. Each dot represents a different variant of a particular gene related to the respective trait.

Our identified hub genes present potential topics for further research, as many of these genes are associated with various diseases observed across multiple traits in genome-wide association studies (GWAS). From Table 2 and Fig. 6, we see that TGFB1 is linked to type 2 diabetes, while BSN is associated with general cognitive ability and Crohn’s disease. RHOC is related to Hypertension, CAMK2A is connected to gut microbiota, and MMP14 has been linked to prostate cancer. Most of these genes display weak associations. However, there are two strong genes: SERPINA1, which is related to sex hormone-binding globulin levels and GABRG2 associated with Femur bone mineral density. We also observed different variants in Fig. 5, which previous studies have associated with multiple traits.

## 4. Discussion

The objective of this study was to identify co-regulatory gene networks predictive of GBM status and one-year survival, while uncovering the protein-level interactions that support these networks. We performed differential gene expression analysis, then applied netLDA to identify strong and weak genes based on statistical significance and correlation structure. This allowed us to detect predictive subnetworks followed by hub genes within PPI networks, providing insight into pathways relevant to GBM and traits identified through GWAS. Unlike traditional approaches that prioritize only highly DE genes, our model considers marginally informative, and non-informative genes, recognizing their potential contributions through co-regulation. We additionally compared the model-based survival separation against single-gene approaches to highlight the added value of network integration. Specifically, our approach began with differential gene expression analysis, followed by pathway enrichment analysis to assess the biological relevance of genes selected for modeling. Among the top five enriched pathways, four were common to the candidate gene sets of both models, namely Parkinson’s Disease, Huntington’s Disease, neurodegeneration pathways, and Alzheimer’s Disease. We found that Parkinson’s disease and GBM share a key factor named Neurotrophic factor, which plays a pivotal role in neurodegeneration and tumor progression [20]. Additionally, some proteins implicated in Huntington’s disease, such as mutated huntingtin-interacting protein 1 (HIP1), have been shown to correlate with elevated epidermal growth factor receptor (EGFR) signaling, a hallmark of aggressive GBM [21]. Neurodevelopment pathways also play a vital role in brain tumors, and the transcriptome associations between Alzheimer’s disease and GBM were also explored before [22]. Therefore, the set of genes used to select predictive networks of strong and weak genes in the netLDA model was found to be related to brain disease, specifically GBM.

In our disease prediction model, GABRA6 emerged as the leading strong gene. Although its precise role in GBM remains unclear, recent research has begun to explore its association with glioma [23]. Notably, GABRA6 shows limited protein-level interactions but may be linked to the unfolded protein response (UPR), a pathway critical to proteostasis and known to influence growth, invasion, resistance, and angiogenesis in GBM [24]. A previous study also showed its connection with one of the top enriched pathways, which is Huntington’s disease [25]. Our analysis underscores the value of network-based approaches by identifying hub genes with extensive protein-level interactions. For instance, BSN emerged as a hub in the largest network and has been previously recognized as a key gene in GBM studies [26]. In network 26, some weak genes including RHOC and annexin family members ANXA2 and ANXA1, all of them are prognostically significant in GBM [27, 28], were also highlighted. For the one-year survival prediction model, LAIR1 emerged as the top weak gene and was inversely correlated with the survival of GBM patients [29]. Notably, SERPINA1, the only strong gene in a highly predictive network, has been documented to have links to glioma [30]. Additionally, we observed high connectivity among several hub genes within these networks; for example, ITGAM, a weak gene in network 3, aligns with integrin-based prognostic signatures described in glioma research [31]. Many of our top-ranked differentially expressed genes (DEGs), including NRGN and BSN, are functionally implicated in synaptic signaling and neuronal maintenance—processes also central to the pathology of Parkinson’s and Huntington’s diseases. The enrichment of these genes in overlapping pathways suggests that disruptions in synaptic plasticity and cellular stress responses may be common drivers in both tumorigenesis and neurodegeneration, albeit with divergent outcomes: hyperproliferation in GBM versus neuronal loss in Parkinson’s and Huntington’s disease.

The study revealed minimal overlap between the strong and weak gene sets in the disease prediction and survival prediction models, indicating that different genetic pathways may contribute to the onset of the disease and its progression. Only two genes, CHI3L2 and CD163, were common in the strong gene set, while the weak gene sets had seven common genes: MOBP, PLP1, MBP, CTSS, HAVCR2, ERMN, and HLA-DMA. CHI3L2 has been associated with poor immune cell infiltration in gliomas, while CD163 is a marker of M2 macrophages and plays a role in the progression of Glioblastoma Multiforme (GBM) by promoting tumor growth and suppressing the immune response [32, 33]. Among the weak genes, MOBP, ERMN, and PLP1 have been identified as specific biomarkers associated with the progression of GBM [34]. Additionally, CTSS was linked to tumor progression in a recent study. The study also found that hub genes are associated with diverse traits, indicating genes’ pleiotropic roles. ANXA1, a weak gene, has been detected with loss of expression in multiple tumors. Furthermore, some hub genes, such as CTSS and HAVCR2, have vital multiple traits reported in other studies, suggesting their broader implications in health and disease. In 1985, MBP was studied to understand its relationship with different types of tumors [35]. In other studies, HAVCR2 was reported as potential biomarkers pan-cancer analysis [36]. A high level of CTSS was related to tumor progression in a recent study [37]. From the above findings, it is apparent that the strong genes that overlap and the associated less-expressed weak genes, as identified by both models using our network-based machine learning approach, were important for GBM progression, survival, and immune response-related studies.

Based on the genes we highlighted above, the insights from Genome-wide association studies (GWAS) added another layer of understanding. After being screened for significant survival, the hub genes were associated with diverse traits. This underpins genes’ pleiotropic roles, mirroring findings from other genomic studies [38]. For example, there is a weak gene, ANXA1, for which loss of expression has been detected in multiple tumors [39]. We can also see some overlapping hub genes, both strong and weak, that have vital multiple traits reported in other studies. One of the weak genes, CTSS, related to renal function, is a targeted gene for the study of renal diseases, such as kidney disease [40]. Previous studies showed the risk of developing cancers like skin cancer after kidney disease because of immune dysfunction [41, 42]. Notably, this gene, CTSS, was also found to be associated with basal cell carcinoma [43]. Therefore, targeting this gene can be a potential therapeutic approach for studying disease interconnections. Another strongly overlapping connected hub gene, HAVCR2, is related to Hepatitis A virus cellular receptor 2 levels [44]. Such observations highlight the multifaceted roles of these genes and suggest their broader implications in health and disease.

Overall, this study presents an applied integrative machine learning framework that combines transcriptomic network discovery with protein–protein interaction analysis to predict glioblastoma (GBM) disease status and one-year survival. By leveraging gene–gene co-regulation patterns, our approach identifies biologically meaningful gene networks and reveals interactions that may not be captured by conventional PPI-based methods alone. While the survival prediction performance (AUC ≈ 0.80) is encouraging, the model is not yet suited for clinical use. Instead, these findings serve as hypothesis-generating results that highlight candidate genes and regulatory modules for follow-up mechanistic and clinical validation. Some of the other limitations include the absence of socio-demographic covariates, possibility of overfitting and focus on only on transcriptomics data. In future works we aim to incorporate multi-omics integration, independent validation, and additional hub-selection criteria to enhance prognostic accuracy and biological interpretability. Nonetheless, this work establishes a foundation for the development of network-driven predictive models in oncology research.

## Supplementary Material

MMC1

MMC2

MMC3

MMC4

MMC5

MMC6

MMC7

MMC13

MMC12

MMC9

MMC10

MMC11

MMC8

## Figures and Tables

**Table 1:** Predictive gene networks for both models.

Disease/Non-disease Model			

Network	Total genes	Strong gene	Weak genes
Network 2	36	FAM153B, KCNT1, GLS2, HOOK1, CKMT1B, CNNM1, ATP8A2, CACNA1I, GRIN1, CARMIL2, CORO6, JPH3, SCRT1, MIR124–1HG, SLC8A2, WSCD2, KSR2, FGF17, CACNA1B	SLC12A5, ARHGAP44, ABCG4, PHYHIP, STX1B, CKMT1A, NEURL1, CPLX1, BSN, BICDL1, ADAM11, UNC5A, RIMS1, PRRT2, PRRT1, LOC100507547, CBFA2T3
Network 26	33	MMP2	CMTM3, FCGRT, ATP6V0E1, TGFB1, RHOC, GNG5, IGFBP7, LRP10, FKBP10, ANO6, ARPC1B, RAB13, ANXA2, ANXA1, S100A10, MSN, MMP14, IFI16, MAGT1, CLIC4, PARP4, LIMS1, LITAF, SYPL1, RDX, SEC11A, CBFB, SPPL2A, HDAC1, CD99, ABHD4, DDR1
Network 15	23	PRKCG	DLGAP3, FBXL16, PSD, DMTN, KCNIP2, KCNH3, SEPTIN5, KCNAB2, PNCK, AGAP2, KCNQ2, PDZD4, DUSP8, RAPGEFL1, SNPH, PDXP, FAM189A1, LMTK3, ABHD8, FBXL19, RTN2, PHF24
Network 21	23	CACNG3, LRTM2	CAMK1G, SH2D5, CAMKV, PTPN5, CAMK2A, MATK, FXYD7, KCNV1, CHRM1, CREG2, KCTD16, NRGN, CACNG8, KCNN1, GRIN2B, GABRA5, FGF13, VSTM2A, RBP4, LY6H, DDN
Network 12	20	SYNPR, GABRG2	ST8SIA3, RGS7, SNAP25, SCN2B, RAB3A, UNC13A, NAP1L2, CAMKK1, GLT1D1, TUBA8, GABRB3, ACTL6B, L1CAM, SYT4, ATP1A3, INA, SYT7, DGCR5
Network 28	19	TMEM235	NKX6–2, MAG, KLK6, TMEM125, MYRF, CNDP1, CAPN3, PLP1, TMEM144, ERMN, S1PR5, CLDN11, MBP, MOBP, MTURN, SHTN1, MAL, HHATL
Network 20	10	CHD5	SHANK1, PNMA3, PNMA6A, PRKAR1B, GNG3, BEX5, MAPK8IP2, ARHGDIG, CABP1
Network 17	8	MSR1, CD68	HLA-DPB1, HLA-DPA1, HAVCR2, HLA-DMA, CYBB, CTSS
Network 18	5	GABRA6	CRTAM, PDZK1, CDC42BPG, PCSK9
**1-year survival Model**			
Network 41	26	SERPINA1	LAIR1, HCK, SPI1, FERMT3, CYTH4, FCGR3A, SYK, CD68, PTPN6, HAVCR2, GPSM3, MYO1F, TBXAS1, CSF1R, EVI2B, IL10RA, TLR2, ADORA3, HLA-DMA, CTSS, MS4A7, ITGAM, DOCK2, FCGR2A, MS4A4A
Network 3	6	CNDP1, MAG	MBP, MOBP, PLP1, ERMN

**Table 2:** Multiple traits for the Hub genes identified from GWAS, along with their number of associated studies.

Gene	Type of gene	Traits	Associated Studies

BSN	Weak	Intelligence (MTAG), General cognitive ability, Intelligence, Cognitive function	9
		Crohn’s disease	5
TGFB1	Weak	Coronary artery disease	10
		Osteoarthritis	6
MMP14	Weak	Prostate cancer	4
RHOC	Weak	Systolic blood pressure	2
ANXA1	Weak	Brain region volumes	3
ANXA2	Weak	Annexin A2 levels	8
		Type 2 diabetes	2
CAMK2A	Weak	Gut microbiota (bacterial taxa)	4
GABRG2	Strong	Femur bone mineral density x serum urate levels interaction	3
MAG	Weak	Mean corpuscular hemoglobin concentration	2
ITGAM	Weak	IgA nephropathy	7
SERPINA1	Strong	Sex hormone-binding globulin levels	35
CTSS	Weak	Renal function	1
SYF	Weak	Platelet count	6
FCGR3A	Weak	leukocyte count	31
HAVCR2	Weak	Hepatitis A virus cellular receptor 2 levels	7

## Data Availability

The Cancer Genome Atlas (TCGA), Genotype-Tissue Expression (GTEX), and this can be downloaded from the following website http://xena.ucsc.edu/.

## Appendix A. Supporting information

Supplementary data associated with this article can be found in the online version at doi:10.1016/j.ejca.2025.116197.
