# Supplementary material for: Integrative machine learning reveals hidden and emerging co-regulatory gene networks for multi-phase glioblastoma outcome prediction
Source: Eur J Cancer. Author manuscript; Available in PMC 2026 Aug 10. (PMC13454984; doi:10.1016/j.ejca.2025.116197)
Supplement: Supplementary file 1 [file NIHMS2189826-supplement-MMC2.docx]

**Supplementary Figure 1: Detailed Workflow Steps for Data Selection Process.**

**Supplementary Figure 2: Overall model performance was evaluated using the Receiver Operating Characteristic (ROC) for both models, along with the Area Under the Curve (AUC): (**A) Disease/Non-disease analysis and (B) 1-year survival analysis.

**Supplementary Figure 3 (A)-(B): Heatmap illustrating the gene expression analysis for both disease and non-disease categories.** The color gradient in the heatmap indicates variations in expression levels.

**Supplementary Figure 4: Heatmap Representation of Gene Expression Patterns from the One-Year Survival Analysis**. This figure illustrates the relative expression levels of selected genes that were significantly associated with survival outcomes over one year. The color gradient in the heatmap indicates variations in expression levels, allowing for easy comparison of gene activity across different sample groups. Highlighted genes may show potential prognostic value in assessing patient survival.

**Supplementary Figure 5: Overlapping Genes Between Two Models.** In (A), we represent strong genes, and in (B), we illustrate weak genes.
